# Russian adaptation of Stress Mindset Measure-General (SMM-G)

**DOI:** 10.1192/j.eurpsy.2022.899

**Published:** 2022-09-01

**Authors:** N. Lebedeva, E. Solenova

**Affiliations:** 1 Moscow Metropolitan Governance University, Diagnostics Department, Moscow, Russian Federation; 2 Lomonosov Moscow State University, Faculty Of Psychology, Moscow, Russian Federation

**Keywords:** mindset, Stress, scale

## Abstract

**Introduction:**

The 8-item Stress Mindset Measure-General (SMM-G) is an instrument designed to assess stress-is-enhancing and stress-is-debilitating mindsets. The stress-is-enhancing stress mindset positively correlates with well-being indices and work productivity and negatively correlates with depression and anxiety scores. Mindset could be changed after a psychological, psychoeducational, or psychotherapeutic intervention.

**Objectives:**

We aim to adapt the SMM-G for adolescents and to explore its factor structure and psychometric properties in a sample of Russian students.

**Methods:**

A total of 564 Russian students (337 men, 229 women) from 9 universities aged 17 to 23 years (М=19,9) participated in the study. We computed reliability indicators, conducted exploratory factor analysis (EFA) and confirmatory factor analysis (CFA).

**Results:**

Psychometric indicators are shown in Table 1. As a result of EFA (maximum likelihood, varimax rotation), two factors (eigenvalues 3,430 and 1,645) were extracted, accounting for 42,9% and 20,6% explained variance. Then, we tested the proposed model via CFA (Table 2).

**Table 1.** Psychometric indicators

Mean value ± std	Cronbach`s alpha	Test-retest reliability (n=179, one month between assessments)
1,175±0,165	0,805	0,563

The first factor includes all odd-numbered questions, while the second factor contains all even-numbered questions. This is consistent with the questionnaire’s structure, leading to a natural interpretation of factors as the stress-is-enhancing and stress-is-debilitating mindsets.

**Table 2.** CFA results

Fit indices	Acceptable values	One-Factor Model	Two-Factor Model
CMIN/DF	≤5	7,516	*4,298*
GFI	≥0,9	0,895	*0,959*
AGFI	≥0,9	0,828	*0,902*
CFI	≥0,9	0,680	0,890
RMSEA	≤0,08	0,107	*0,076*

**Conclusions:**

Russian adaptation of SMM-G has shown good psychometric characteristics and constitutes a useful assessment instrument.

**Disclosure:**

No significant relationships.

